# Supplementary material for: Structural insights into opposing actions of neurosteroids on GABAA receptors
Source: Nat Commun. 2023 Aug 22;14:5091. doi: 10.1038/s41467-023-40800-1 (PMC10444788; doi:10.1038/s41467-023-40800-1)
Supplement: Supplementary file 1 — Supplementary Information [file 41467_2023_40800_MOESM1_ESM.pdf]

Supplementary Information

Structural insights into opposing actions of neurosteroids on $GABA_A$ receptors

Dagimhiwat H. Legesse[1], Chen Fan[2], Jinfeng Teng[3], Yuxuan Zhuang[2], Rebecca J. Howard[2], Colleen M. Noviello[3], Erik Lindahl[2,4,]* and Ryan E. Hibbs[1,3,]*

Affiliations:

[1]Department of Neuroscience, UT Southwestern Medical Center, Dallas, TX, USA

[2]Dept. of Biochemistry and Biophysics, Science for Life Laboratory, Stockholm University, Solna, Sweden

[3]Department of Neurobiology, University of California San Diego, La Jolla, CA, USA

[4]Dept. of Applied Physics, Science for Life Laboratory, KTH Royal Institute of Technology, Solna, Sweden

*Correspondence:

Erik Lindahl, erik.lindahl@scilifelab.se

Ryan E. Hibbs, rehibbs@ucsd.edu

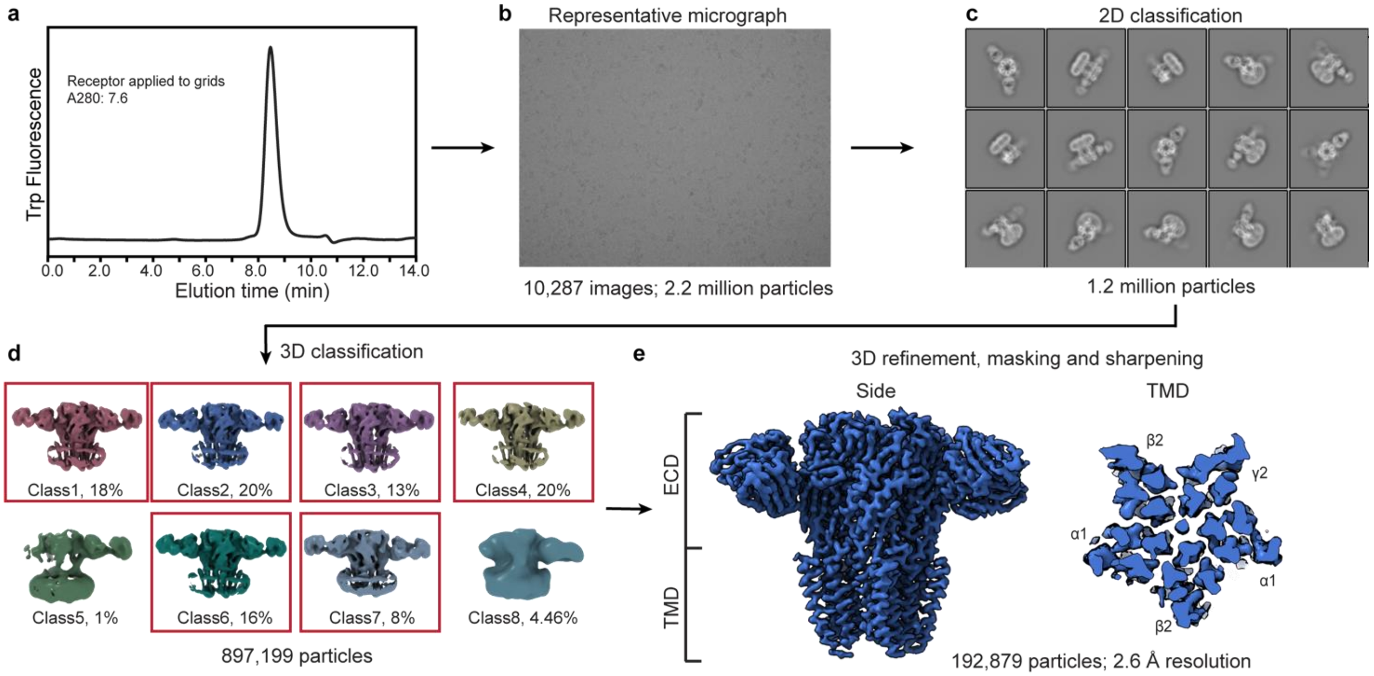

Supplementary Figure 1: Cryo-EM processing of GABA$_A$ receptor-pregnenolone sulfate complex. a, Size-exclusion chromatogram of PS bound complex in brain lipid nanodisc. **b,** A representative cryo-EM micrograph. **c**, 2D classification. **d**, 3D classification results, colored arbitrarily by class. Selected classes are boxed in red. **e**, Side and TMD top view of final reconstruction.

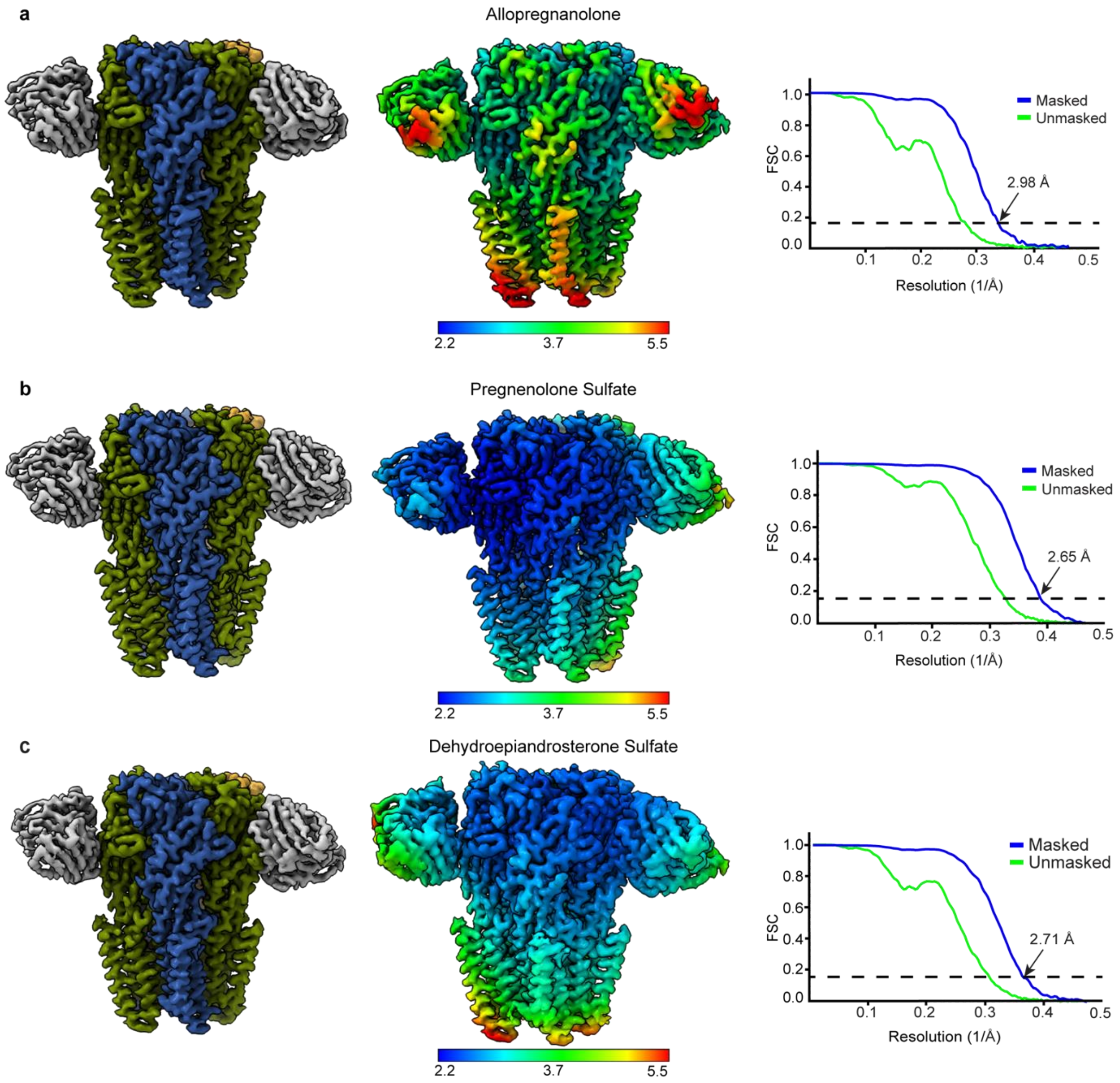

Supplementary Figure 2: Local and global resolution estimates. a, Allopregnanolone overall map quality, colored by local resolution estimated by Relion, and global resolution at FSC = 0.143. **b**, As in **a** but for PS dataset. **c**, As in **a** but for DHEAS dataset.

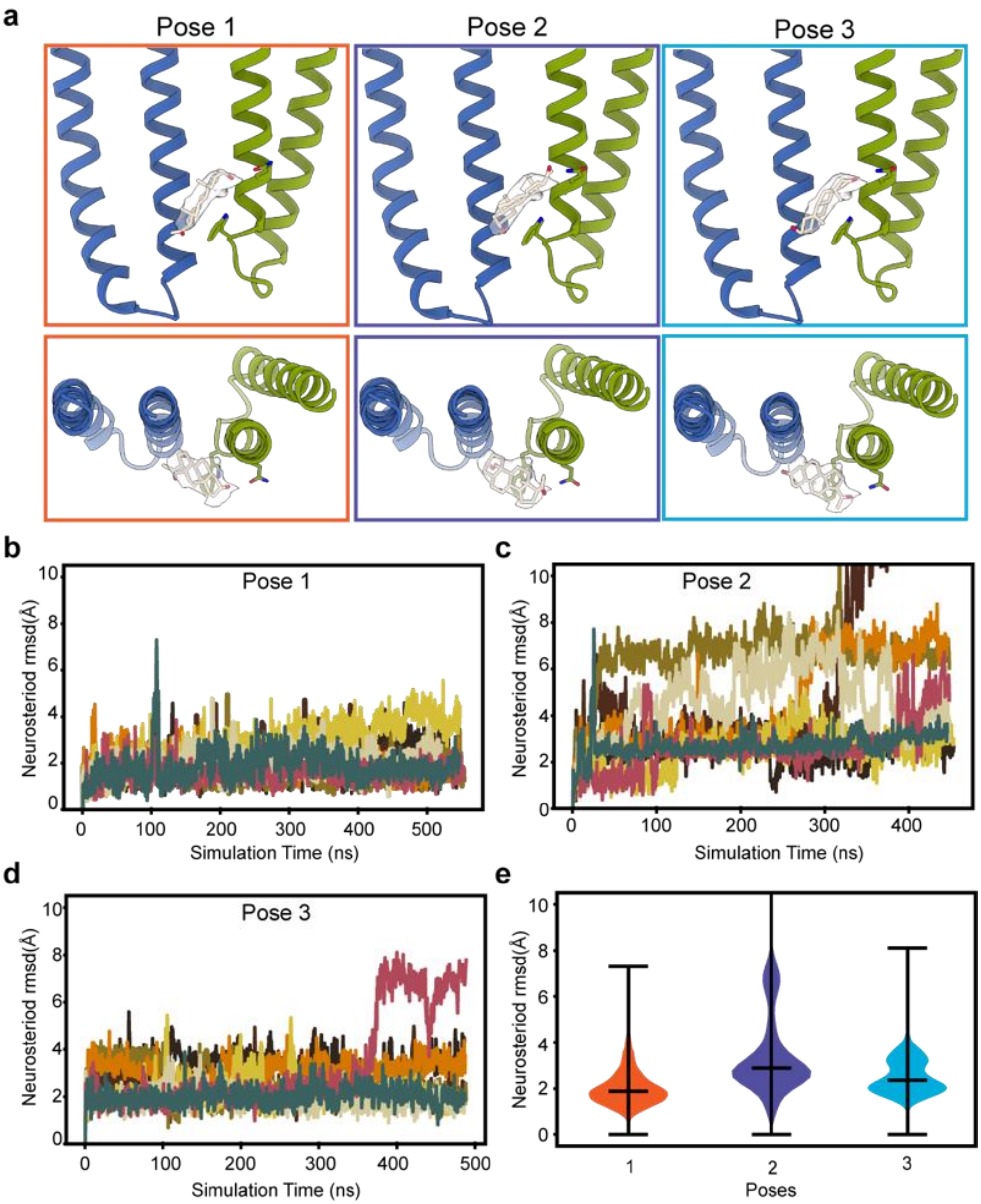

Supplementary Figure 3: MD simulations of allopregnanolone in different poses. a, Illustration of the three allopregnanolone poses tested in MD simulations. The β2 and α1 subunits are colored blue and green. The density for allopregnanolone is shown as a transparent surface, and three different poses of allopregnanolone are shown as sticks, colored in wheat. **b-d**, Allopregnanolone rmsd during simulations initiated in poses 1–3. The results from different simulations (4 replicates * 2 binding sites) are colored differently. **e**, Comparison of allopregnanolone binding stability in poses 1–3 during simulations. Violin plots represent probability densities from 4 independent simulation replicates of >400 ns each, sampled every 0.4 ns (n > 4000), with markers indicating median and extrema.

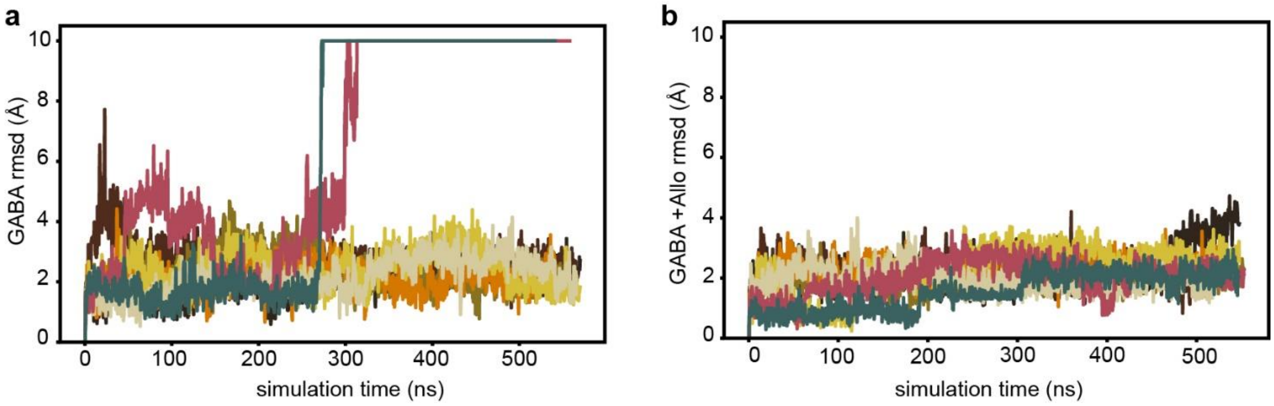

Supplementary Figure 4: Allopregnanolone influences GABA stability. a–b, Time courses for GABA rmsd in simulations of the GABA-only (**a**) and GABA + allopregnanolone (**b**) systems. Results from different simulations (4 replicates * 2 binding sites) are colored differently.

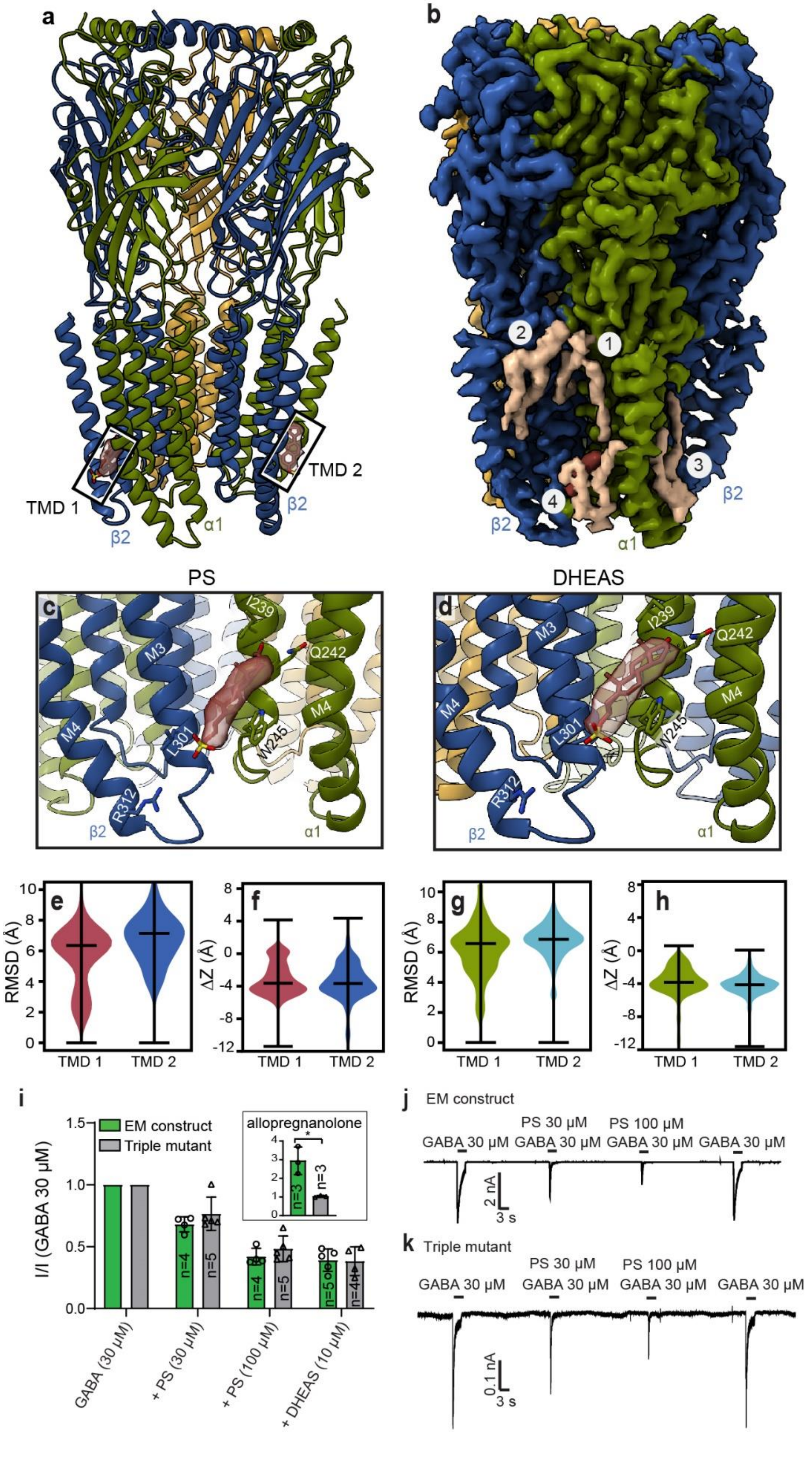
a
b
TMD 1
TMD 2
β2
α1
1
2
3
4
PS
DHEAS
c
d
I239
Q242
M3
M4
L301
W245
R312
e
f
g
h
RMSD (Å)
ΔZ (Å)
TMD 1
TMD 2
i
I/I (GABA 30 μM)
EM construct
Triple mutant
allopregnanolone
n=3
n=4
n=5
GABA (30 μM)
+ PS (30 μM)
+ PS (100 μM)
+ DHEAS (10 μM)
j
EM construct
PS 30 μM
PS 100 μM
GABA 30 μM
2 nA
3 s
k
Triple mutant
0.1 nA

Supplementary Figure 5: Study of potential site for sulfated steroids in PAM neurosteroid locus. a and **b**, Structural overview of DHEAS model and map illustrating four classes of lipid sites. MD simulations in later panels refer to the TMD sites 1 and 2 in panel **a**. **c** and **d** show details of representative density in the PAM neurosteroid site for the PS and DHEAS structures, respectively. **e**, PS stability based on rmsd in simulations initiated in TMD pose 1 (red) and pose 2 (blue). **f**, PS movement along the channel pore (z-axis) during simulations for the two TMD poses. PS moves toward the inner leaflet of the membrane (negative) in both poses. **g,h**, Binding and movement analyses as in panels **e-f** for DHEAS in TMD pose 1 (green) and pose 2 (cyan). Violin plots (e–h) represent probability densities from 4 independent simulation replicates of >400 ns each, sampled every 0.4 ns (n > 4000), with markers indicating median and extrema. **i**, Summary of electrophysiology data comparing effects from NAM neurosteroids on GABA currents in the EM construct and in the triple mutant designed to knock out the major interactions in the PAM site. Inset is the response from allopregnanolone showing that the triple mutant does knock out activity of the PAM. Results are presented as normalized peak currents. Replicate numbers from independent cells are indicated in each bar. Statistical analysis was performed using GraphPad Prism 9.2.0 software (GraphPad software, Inc, La Jolla, CA). Data are expressed as means ± S.D. The two-tailed Welch's t-test was used. A p value of ≤0.05 (*) was considered statistically significant. **j** (EM) and **k** (triple mutant) are representative recordings used to generate the bar graph in **i**.

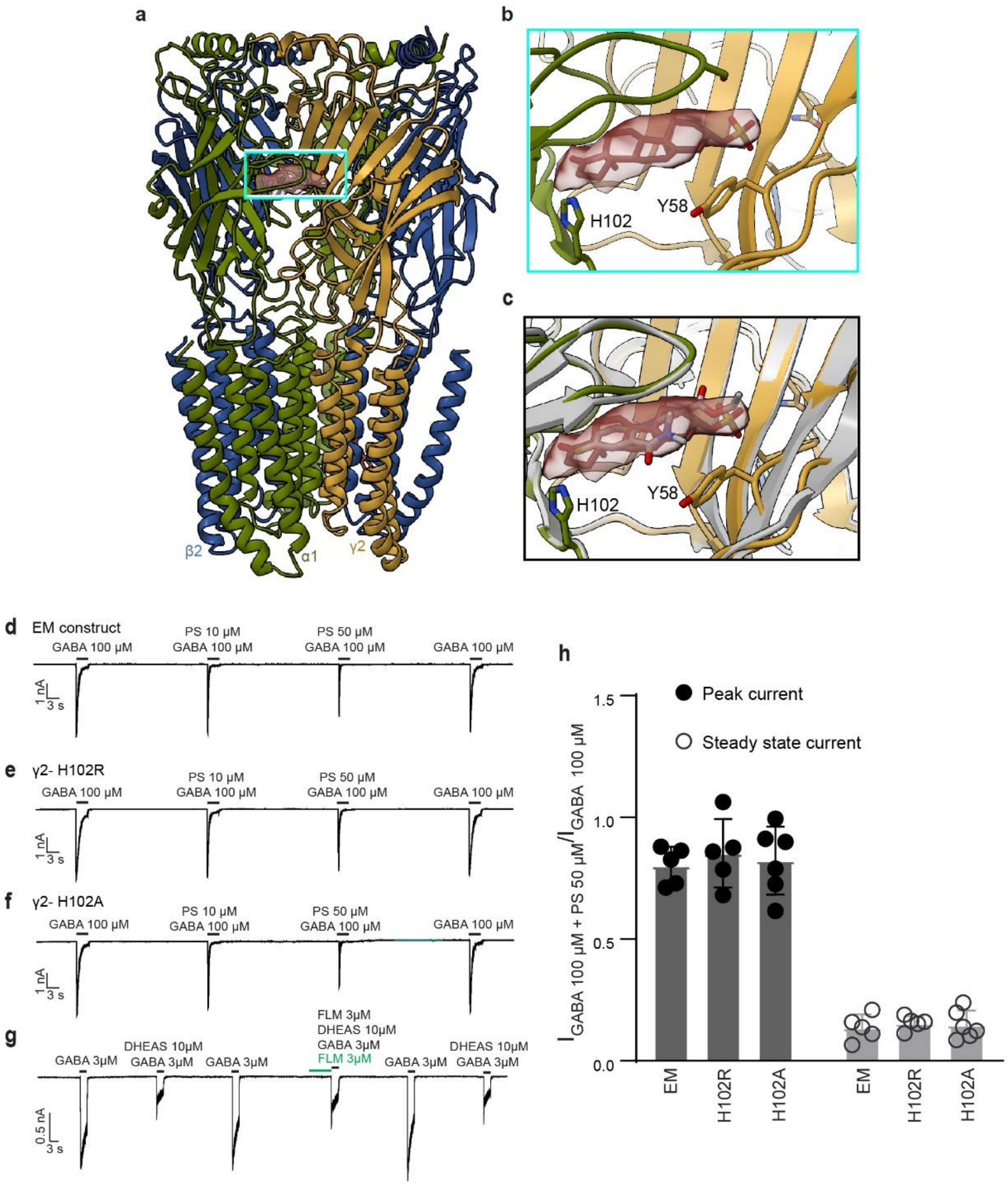

Supplementary Figure 6: Testing relevance of sulfated neurosteroid binding in the ECD benzodiazepine site. a, Structural overview highlighting location of benzodiazepine site at α-γ interface where extra density was observed in PS and DHEAS structures. Subunits are colored as in Fig. 2. **b**, Detail for DHEAS in scarlet with density shown as a semitransparent surface. **c**, Same as **b**, but with flumazenil (grey) bound as it is in PDB 6X3U, illustrating how flumazenil would directly compete with binding where this extra density is observed. **d**-**g**, Whole cell patch-clamp electrophysiology recordings of the EM construct and mutants thereof in the benzodiazepine site, as well as tests with flumazenil, illustrating that knocking out this site has no effect on NAM neurosteroid activity. Recordings are from n ≥ 3 independent cells for each condition yielding similar results. **h**, Comparison of PS inhibition of the peak and steady state currents of EM construct and mutants in the EM construct background. The bars indicate mean current ± SD, n = 5 (EM), 5 (H102R), and 6 (H102A) recordings from individual cells.

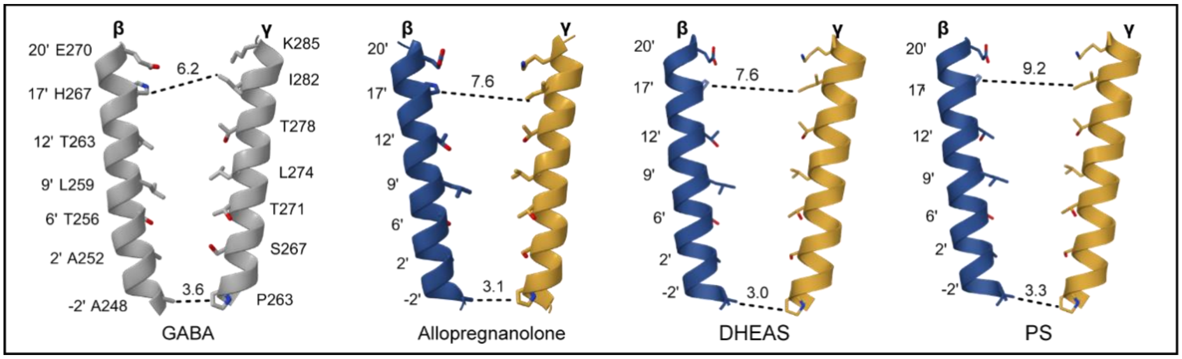

Supplementary Figure 7: Conformational differences in the pore stabilized by neurosteroids. M2 helices are shown for β and γ subunits with analysis and colored as in Fig. 3e-h. Numbers indicate pore diameters in Å at different positions. PDB code for GABA alone: 6X3Z.

Supplementary Table 1. Cryo-EM data collection, refinement, and validation statistics.

	Allopregnanolone + GABA	Dehydroepiandrosterone Sulfate + GABA	Pregnenolone Sulfate + GABA
**PDB/EMDB Accession Codes**	8SI9 / EMD-40503	8SID / EMD-40506	8SGO / EMD-40462
**Magnification**	81K	81K	81K
**Voltage (kV)**	300	300	300
**Electron exposure (e-/ Å^2)**	50	50	50
**Pixel size (Å)**	1.070	1.068	1.069
**Symmetry imposed**	C1	C1	C1
**Initial particle images (no.)**	1,460,740	2,203,137	1,414,490
**Initial particle images (no.)**	167,534	188,416	367,532
**Map Resolution (Å)**	2.98	2.71	2.65
**FSC threshold**	0.143	0.143	0.143
**Map Resolution Range (Å)**	2.7-5	2.2-4.5	2.2-4.5
**Refinement**			
**Initial model used (PDB code)**	6X3V	6X3V	6X3V
**Model resolution (Å)**			
**FSC threshold**	0.5	0.5	0.5
**Map-sharpening B-factor (Å^2)**	-15	-15	-15
**Model composition**			
**Nonhydrogen atoms**	17204	17,465	17,210
**Protein residues**	2,119	2,121	2,121
**Ligand**	4	3	3
**B-factor (Å^2)**			
**Protein**	43.21	33.35	33.88
**Ligand**	48.04	40.20	40.67
**Rmsd values**			
**Bond length (Å)**	0.005	0.007	0.005
**Bond angles (Å)**	0.620	0.612	0.512
**Validation**			
**Molprobity score**	1.46 (100%)	1.44 (100%)	1.61 (100th %)
**Clashscore**	3.61 (100%)	5.5 (97%)	5.24 (100th %)
**Poor rotamers (%)**	0.0	0.00	0.00
**Ramachandran analysis**			
**Favored (%)**	95.38	95.58	97.58
**Allowed (%)**	4.62	4.42	4.35
**Outliers (%)**	0.00	0.00	0.00

Supplementary Data Table 2. Molecular dynamics simulation parameters.

	GABA only	GABA+Allo	GABA+PS	GABA+DHEAS
**Simulation box**	127Å x 127Å x 162Å	127Å x 127Å x 163Å	127Å x 127Å x 163Å	127Å x 127Å x 163Å
**Number of atoms**	272491	272934	272600	272392
**Number of waters**	61939	62056	61957	61889
**Salt concentration**	150mM NaCl	150mM NaCl	150mM NaCl	150mM NaCl
**Number of lipids**	440 POPC	440 POPC	440 POPC	440 POPC
