# Supplementary material for: Structural insights into opposing actions of neurosteroids on GABAA receptors
Source: Nat Commun. 2023 Aug 22;14:5091. doi: 10.1038/s41467-023-40800-1 (PMC10444788; doi:10.1038/s41467-023-40800-1)
Supplement: Supplementary file 3 — Reporting Summary [file 41467_2023_40800_MOESM3_ESM.pdf]

nature portfolio

Corresponding author(s): Ryan Hibbs and Erik Lindahl

Last updated by author(s): 8/02/2023

Reporting Summary

Nature Portfolio wishes to improve the reproducibility of the work that we publish. This form provides structure for consistency and transparency in reporting. For further information on Nature Portfolio policies, see our Editorial Policies and the Editorial Policy Checklist.

Statistics

For all statistical analyses, confirm that the following items are present in the figure legend, table legend, main text, or Methods section.

n/a	Confirmed	
[ ]	[x]	The exact sample size (*n*) for each experimental group/condition, given as a discrete number and unit of measurement
[ ]	[x]	A statement on whether measurements were taken from distinct samples or whether the same sample was measured repeatedly
[x]	[ ]	The statistical test(s) used AND whether they are one- or two-sided<br>*Only common tests should be described solely by name; describe more complex techniques in the Methods section.*
[x]	[ ]	A description of all covariates tested
[x]	[ ]	A description of any assumptions or corrections, such as tests of normality and adjustment for multiple comparisons
[ ]	[x]	A full description of the statistical parameters including central tendency (e.g. means) or other basic estimates (e.g. regression coefficient) AND variation (e.g. standard deviation) or associated estimates of uncertainty (e.g. confidence intervals)
[x]	[ ]	For null hypothesis testing, the test statistic (e.g. *F*, *t*, *r*) with confidence intervals, effect sizes, degrees of freedom and *P* value noted<br>*Give P values as exact values whenever suitable.*
[x]	[ ]	For Bayesian analysis, information on the choice of priors and Markov chain Monte Carlo settings
[x]	[ ]	For hierarchical and complex designs, identification of the appropriate level for tests and full reporting of outcomes
[x]	[ ]	Estimates of effect sizes (e.g. Cohen's *d*, Pearson's *r*), indicating how they were calculated

Our web collection on statistics for biologists contains articles on many of the points above.

Software and code

Policy information about availability of computer code

Data collection	pCLAMP 11
Data analysis	Relion 3.1, Phenix 1.19-4092, UCSF Chimera 1.15 (build 42258), UCSF ChimeraX 1.5, GraphPad Prism 9, Molprobity 4.5.2, Coot 0.9.4.1, Hole2, SBGrid, MotionCor2, GCTF, CrYOLO 1.5.6, CHARMM-GUI, CGenFF, GROMACS 2020.5 and 2021.5, Matplotlib, VMD, ProLIF 1.1.0.

For manuscripts utilizing custom algorithms or software that are central to the research but not yet described in published literature, software must be made available to editors and reviewers. We strongly encourage code deposition in a community repository (e.g. GitHub). See the Nature Portfolio guidelines for submitting code & software for further information.

Data

Policy information about availability of data

All manuscripts must include a data availability statement. This statement should provide the following information, where applicable:

- Accession codes, unique identifiers, or web links for publicly available datasets
- A description of any restrictions on data availability
- For clinical datasets or third party data, please ensure that the statement adheres to our policy

The cryo-EM maps have been deposited in the Electron Microscopy Data Bank (EMDB) under accession codes EMD-40503 [https://www.ebi.ac.uk/pdbe/entry/emdb/EMD-40503] (GABA + allopregnanolone); EMD-40462 [https://www.ebi.ac.uk/pdbe/entry/emdb/EMD-40462] (GABA + pregnenolone sulfate); and EMD-40506 [https://www.ebi.ac.uk/pdbe/entry/emdb/EMD-40506] (GABA + DHEAS). The atomic coordinates have been deposited in the Protein Data Bank (PDB)

under accession codes PDB-8SI9 [https://doi.org/10.2210/pdb8SI9/pdb] (GABA + allopregnanolone); PDB-8SGO [https://doi.org/10.2210/pdb8SGO/pdb] (GABA + pregnenolone sulfate); and PDB-8SID [https://doi.org/10.2210/pdb8SID/pdb] (GABA + DHEAS). Previously published structures compared in the study include: 6X3Z [http://doi.org/10.2210/pdb6X3Z/pdb], 6X3X [http://doi.org/10.2210/pdb6X3X/pdb], 6X3V [http://doi.org/10.2210/pdb6X3V/pdb], and 6X3T [http://doi.org/10.2210/pdb6X3T/pdb]. MD simulation trajectory, parameter files, and analysis scripts are available in Zenodo (DOI: 10.5281/zenodo.7770004). The source data underlying Figures 3i,k,l, 5a-c,e-i,j, and 6h, and Supplementary Figures 3e, and 5e-h are provided as a Source Data file.

Human research participants

Policy information about studies involving human research participants and Sex and Gender in Research.

Reporting on sex and gender	N/A
Population characteristics	N/A
Recruitment	N/A
Ethics oversight	N/A

Note that full information on the approval of the study protocol must also be provided in the manuscript.

Field-specific reporting

Please select the one below that is the best fit for your research. If you are not sure, read the appropriate sections before making your selection.

☒ Life sciences ☐ Behavioural & social sciences ☐ Ecological, evolutionary & environmental sciences

For a reference copy of the document with all sections, see nature.com/documents/nr-reporting-summary-flat.pdf

Life sciences study design

All studies must disclose on these points even when the disclosure is negative.

Sample size	No sample size calculation. Rather, a sufficient number of images and particles were collected during a 48-hour microscope time span to achieve high-resolution reconstructions for all cryo-EM datasets.
Data exclusions	No data were excluded from analyses.
Replication	Electrophysiological data were obtained from a minimum of 3 independent cells, and all attempts at replication were successful. Exact numbers of cells (replicates) are specified in the legend of each figure or within the figure itself.
Randomization	The particles selected for all cro-EM data sets were randomly split when estimating the overall resolution. In other experiments, samples were not randomized because it is not technically or practically feasible for electrophysiology and MD experiments. Covariant control was also not achievable due to the need to transfect cells with predetermined amounts and ratios of DNAs.
Blinding	Researchers were not blinded. Blinding is not financially or practically achievable with cryo-EM data collections or electrophysiology experiments.

Reporting for specific materials, systems and methods

We require information from authors about some types of materials, experimental systems and methods used in many studies. Here, indicate whether each material, system or method listed is relevant to your study. If you are not sure if a list item applies to your research, read the appropriate section before selecting a response.

Materials & experimental systems

n/a	Involved in the study	
☐	☒	Antibodies
☐	☒	Eukaryotic cell lines
☒	☐	Palaeontology and archaeology
☒	☐	Animals and other organisms
☒	☐	Clinical data
☒	☐	Dual use research of concern

Methods

n/a	Involved in the study	
☒	☐	ChIP-seq
☒	☐	Flow cytometry
☒	☐	MRI-based neuroimaging

Antibodies

Antibodies used	Standard methods were used to generate the 1F4 monoclonal antibody from the Monoclonal Core of the Vaccine and Gene Therapy Institute at the Oregon Health & Science University, as described in the pubmed ID: 29950725.
Validation	To confirm binding, gel filtration and structural analysis were performed, while the sequence was verified by sequencing of the hybridoma line and experimental density map analysis.

Eukaryotic cell lines

Policy information about cell lines and Sex and Gender in Research

Cell line source(s)	HEK293S GnTI- cells were purchased from ATCC (CRL-3022). Sf9 cells were purchased from ATCC (CRL-1711).
Authentication	Purchased from, ATCC, a commercial supplier. Not further tested.
Mycoplasma contamination	Tested negative by ATCC.
Commonly misidentified lines (See ICLAC register)	No commonly misidentified cell lines were used.
